# The effect of celastrol in combination with 5-fluorouracil on proliferation and apoptosis of gastric cancer cell lines

**DOI:** 10.32604/or.2024.047187

**Published:** 2024-06-20

**Authors:** MOHAMMAD-TAGHI MORADI, DHIYA ALTEMEMY, MAJID ASADI-SAMANI, PEGAH KHOSRAVIAN, MARZIYEH SOLTANI, LEILA HASHEMI, AZADEH SAMIEI-SEFAT

**Affiliations:** 1 Medical Plants Research Center, Basic Health Sciences Institute, Shahrekord University of Medical Sciences, Shahrekord, Iran; 2 Department of Pharmaceutics, College of Pharmacy, Al-Zahraa University for Women, Karbala, Iraq; 3 Cellular and Molecular Research Center, Basic Health Sciences Institute, Shahrekord University of Medical Sciences, Shahrekord, Iran

**Keywords:** Gastric cancer, Celastrol, Terpenoid, Cell cycle regulation, Apoptosis, Synergism

## Abstract

**Background:**

Despite the availability of chemotherapy drugs such as 5-fluorouracil (5-FU), the treatment of some cancers such as gastric cancer remains challenging due to drug resistance and side effects. This study aimed to investigate the effect of celastrol in combination with the chemotherapy drug 5-FU on proliferation and induction of apoptosis in human gastric cancer cell lines (AGS and EPG85-257).

**Materials and Methods:**

In this *in vitro* study, AGS and EPG85-257 cells were treated with different concentrations of celastrol, 5-FU, and their combination. Cell proliferation was assessed using the 3-(4,5-dimethylthiazol-2-yl)-2,5-diphenyl tetrazolium bromide (MTT) assay. The synergistic effect of 5-FU and celastrol was studied using Compusyn software. The DNA content at different phases of the cell cycle and apoptosis rate was measured using flow cytometry.

**Results:**

Co-treatment with low concentrations (10% inhibitory concentration (IC10)) of celastrol and 5-FU significantly reduced IC50 (*p* < 0.05) so that 48 h after treatment, IC50 was calculated at 3.77 and 6.9 μM for celastrol, 20.7 and 11.6 μM for 5-FU, and 5.03 and 4.57 μM for their combination for AGS and EPG85-257 cells, respectively. The mean percentage of apoptosis for AGS cells treated with celastrol, 5-FU, and their combination was obtained 23.9, 41.2, and 61.9, and for EPG85-257 cells 5.65, 46.9, and 55.7, respectively. In addition, the 5-FU and celastrol-5-FU combination induced cell cycle arrest in the synthesis phase.

**Conclusions:**

Although celastrol could decrease the concentration of 5-fluorouracil that sufficed to suppress gastric cancer cells, additional studies are required to arrive at conclusive evidence on the anticancer effects of celastrol.

## Introduction

Gastric cancer was responsible for over one million new cancer cases and approximately 769,000 cancer deaths (the fifth leading cancer in terms of incidence and the fourth leading cancer in terms of mortality) in 2020 worldwide. The incidence rate of gastric cancer is twice in men. Gastric cancer is both the leading high level of cancer and cancer deaths among men in South Central Asian countries including Iran, Afghanistan, Turkmenistan, and Kyrgyzstan [1].

Treatments for gastric cancer include chemotherapy, radiotherapy, surgery, immunotherapy, or often a combination of two or more. Currently, chemotherapy is the standard treatment of choice although the therapeutic effects of chemotherapy drugs are not adequately satisfactory and may cause numerous side effects [2]. As a chemotherapy drug, 5-fluorouracil (5-FU) inhibits DNA synthesis and cancer cell death by inhibiting thymidylate synthase and consequently thymidine nucleotide synthesis. 5-FU is one of the most widely used drugs for the treatment of gastrointestinal cancers, yet it is not tolerated by some patients due to unpleasant side effects [2,3]. Therefore, great emphasis has recently been put on research on anticancer drugs that are compatible with the body system and produce comparably fewer side effects, or whose efficacy is increased and/or whose side effects are reduced in combination with available drugs [4,5].

Celastrol, isolated from *Tripterygium wilfordii* belonging to the boxwood family, is a natural active ingredient. The genus Tripterygium is widely used in traditional medicine to treat swelling, fever, chills, ulcers, and inflammation [6]. Celastrol is a pentacyclic triterpenoid from a small class of natural triterpenoids with a wide range of bioactivities [7]. Because of the *in vivo* anti-inflammatory effect of celastrol, the compound is considered an appropriate choice to modulate inflammation and immune responses [8]. This compound plays a substantial role in treating inflammatory and autoimmune diseases such as arthritis and lupus [9,10]. In addition, studies have confirmed the antitumor effects of celastrol in various types of cancers such as breast, prostate, and colon both *in vitro* and *in vivo*, as well as its impacts on the inhibition of cancer cell proliferation and apoptosis [11,12]. Besides that, celastrol is used to treat chronic nephritis, hepatitis, lupus erythematosus, ankylosing spondylitis, and various skin diseases [13]. Furthermore, numerous studies have demonstrated that celastrol leads to the reduction of Crohn’s disease inflammation and cancer cell differentiation in prostate cancer, and even the compound seems to decrease the tumor necrosis factor (TNF)-α and interleukin (IL)6 cytokines by influencing the nuclear factor κB (NF-κB) pathway [14–16].

Because most chemotherapy drugs cause damage to healthy cells and lead to drug resistance, the combination of herbal compounds with chemotherapy drugs has been recommended to reduce their doses, which consequently decreases their side effects [4,5]. Considering that the effectiveness of different drugs may vary across different cancer types and even different cell lines within a particular cancer, this study was conducted to investigate the effect of celastrol in combination with 5-FU in the treatment of metastatic and resistant gastric cancer cell lines, as well as the signaling pathways involved in these effects.

## Materials and Methods

### Materials

In this experimental *in vitro* study, the AGS and EPG85-257 cells were purchased from the Iran Pasteur Institute Cell Bank (Tehran, Iran) and cultured in Roswell Park Memorial Institute (RPMI) 1640 medium (GIBCO, Grand Island, NY, USA) containing 10% fetal bovine serum (FBS; GIBCO, Grand Island, NY, USA), and 1% penicillin/streptomycin (GIBCO, Grand Island, NY, USA) at 37°C with 5% CO_2_.

Celastrol was purchased from Sigma-Aldrich (St. Louis, MO, USA) and dissolved in dimethyl sulfoxide (DMSO; Samchun, Korea) and phosphate-buffered saline (PBS) to obtain a completely homogeneous solution. The final DMSO concentration in the cell culture did not exceed 0.02%. 5-FU (5-FU PhaRes solution for injection at 5 g/100 mL) was procured from Pharma Resources GmbH (Germany).

### Investigation of cell proliferation using MTT assay

To investigate the growth-inhibiting (anti-proliferative) effects of celastrol and 5-FU on AGS and EPG85-257 cell lines, the cells were treated with different concentrations of celastrol, 5-FU, and their combination, and then cell viability was measured using the 3-(4,5-dimethylthiazol-2-yl)-2,5-diphenyl tetrazolium bromide (MTT) assay. For determination of best combination concentration of celastrol and 5-FU, a relative low dose [10% inhibitory concentration (IC10)] of celastrol was combined with 5-FU at different concentrations.

In the MTT assay, each experiment was performed 3–5 times and the cells were poured into a 96-well culture plate. After 24 h, the cells were incubated with different concentrations of celastrol, 5-FU, and their combination for 48 h. The MTT (Sigma-Aldrich, Germany) solution was prepared at 5 mg/mL in serum-free RPMI and after incubation of the cells for an appropriate duration, the cell supernatant was removed and each well was rinsed with 100 μL of serum-free PBS. Next, the MTT solution was diluted with PBS at 1:5 ratio, and then 60 μL of the resulting solution was added to each well and incubated at 37°C for 4 h. Afterward, the contents of the wells were slowly removed and 100 µL of DMSO was added to them; 10 min later, the optical absorbance of the plates was read at 490 nm wavelength. Cell proliferation rates were compared by comparing the optical absorbance of different wells and the control well. Then, the IC50 value was determined using probit regression in the SPSS software.

The synergistic anti-proliferative effect of 5-FU and celastrol was evaluated using a freely available trial version of CompuSyn software (ComboSyn, Inc. NJ, USA). The anti-proliferative effect of 5-FU+celastrol was maintained at 0.5 IC50, 1 IC50, and 2 IC50 (10 + 2, 20 + 4 and 40 + 8 for AGS cells and 6 + 3.5, 12 + 7 and 30 + 15 μM for EPG85-257 cells). The anti-proliferative effects were investigated using the MTT assay as per the above-explained procedure.

### Evaluation of apoptosis by flow cytometry

To investigate apoptosis, cells were cultured in 6-well plates for 24 h. Then, they were incubated for 48 h with the 0.5 IC50s of celastrol and 5-FU, and the 0.5 IC50s of celastrol and 5-FU combination based on the MTT assay results.

The apoptosis rate of cells under the influence of 5-FU, celastrol, and their combination was measured according to the instructions of the apoptosis detection kit (FITC Annexin V) and flow cytometry kit (Partec, Germany). For this purpose, the cells were separated from the bottom of the plates and washed with calcium buffer twice; then, 10 μL of Annexin V was mixed with 100 μL of cells and incubated in the dark on ice for 20 min; afterward, the cells were washed and 10 μL of propidium iodide stain was added. The resulting mixture was incubated in the dark on ice for 10 min, and the samples were analyzed using flow cytometry [17].

### Investigation of DNA content and cell cycle

DNA content at different phases of the cell cycle was determined using BD Cycletest plus DNA reagent kit (BD Biosciences, CA, USA) and flow cytometry after treatment with 5-FU, celastrol, and their combination (at the concentrations used for investigation of apoptosis). To this end, the cells were cultured in 6-well plates for 24 h. Then, they were further incubated for 48 h with the 0.5 IC50s of celastrol and 5-FU, and the 0.5 IC50 of celastrol and 5-FU combination cultured in a 6-well plate and treated with the studied concentrations of celastrol and 5-FU. Cells were washed with cool PBS and fixated with ethanol 70% after centrifugation at 800 rpm. The cells were then left at 20°C until further investigations. To study cell cycle phases, the control and treated samples were dissolved in 20 mg propidium iodide and 20 mg RNase dissolved in 1 ml PBS; afterward, the samples were studied using flow cytometry. The cell population was calculated at the sub-G1, G0/G1, S, and G2/M phases using the device software.

### Data analysis

The Kruskal-Wallis test in SPSS version 20 was used to investigate the relationship between the data. The probit regression in the same software was used to calculate IC50 and IC10. The curves were plotted using GraphPad Prism 5 Demo software. A *p*-value < 0.05 was considered significance level.

## Results

### Growth-inhibiting effect and combination index value of celastrol and 5-FU

**Figure 1:**
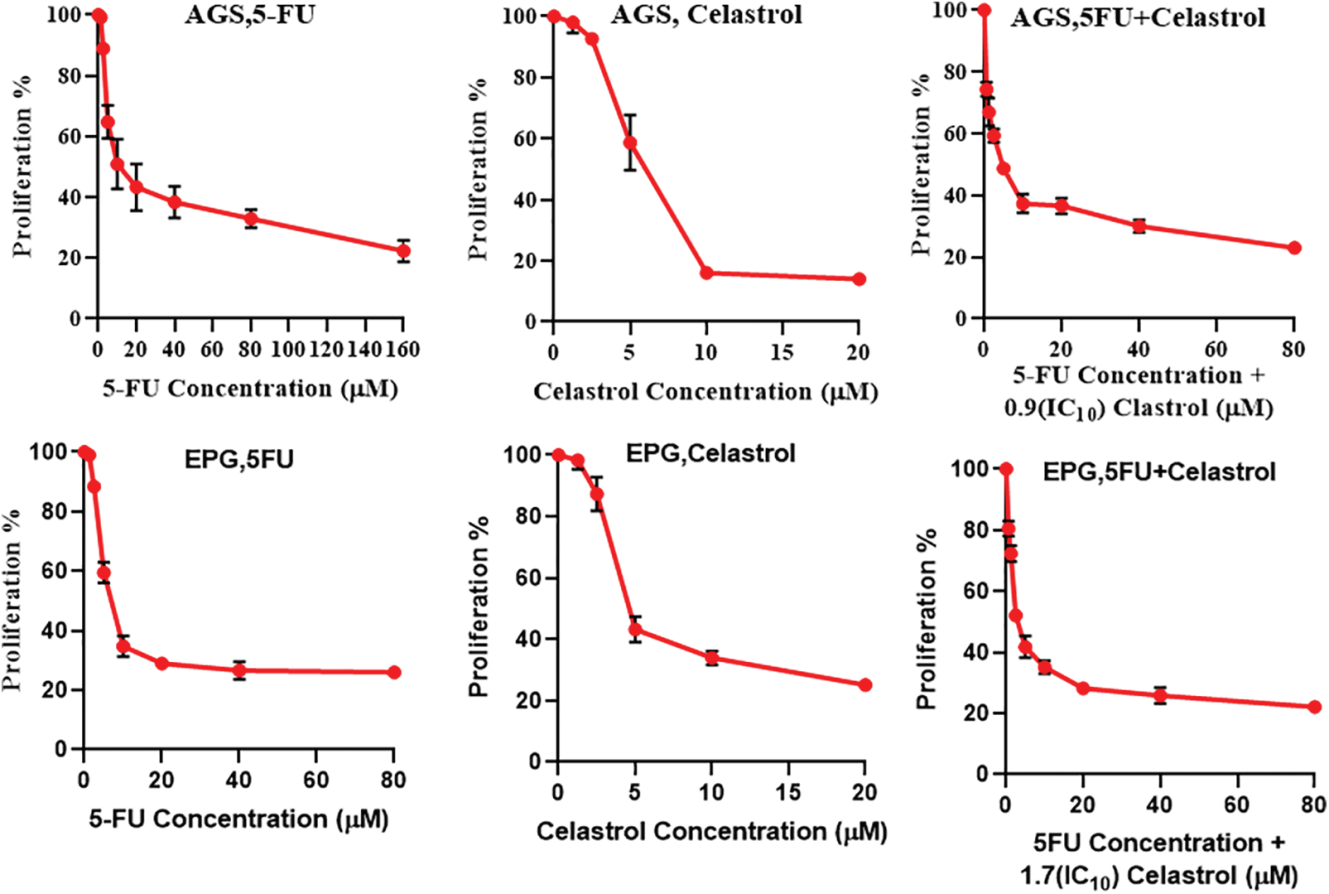
Cells were treated at 48 h with different concentrations of the two compounds separately and their combination, and then cell viability was measured using MTT assay. For determination of the best combination concentration of celastrol and 5-fluorouracil, 10% inhibitory concentration (IC10) of celastrol was combined with 5-FU at different concentrations.

The MTT assay results on cell proliferation showed that cell viability was significantly dependent on the doses of celastrol and 5-FU (*p* < 0.05; Fig. 1). Co-treatment of 5-FU and IC10 of celastrol significantly reduced the IC50 of 5-FU in both cell lines. The IC50 values of the AGS cells treated with 5-FU, celastrol, and their combination were calculated at 20.7, 3.77, and 5.03 μM, and those of the EPG85-257 cells at 11.6, 6.9, and 4.57 μM, respectively (Table 1).

**Table 1:** The IC50 of celastrol, 5-fluorouracil (5-FU), and their combination for studied cell lines

Cell lines Groups	AGS		EPG85-257	
	IC50	CI95%	IC50	CI95%
**5-FU**	20.7	15.7–27.7	11.6	8.5–16
**Celastrol**	3.77	2.86–4.92	6.9	5.5–8.8
**5-FU+Celastrol (IC10)** ^***#***^	5.3*	4.4–6.4	4.57*	3.7–5.6

Note: 5-FU: 5-fluorouracil; IC50: 50% inhibitory concentration; CI95%: confidence interval 95%, #: 10% inhibitory concentration (IC10) of celastrol was combined with 5-FU at different concentrations; *p < 0.05 compared to treatment with celastrol and 5-FU alone.

The synergistic action of celastrol and 5-FU was confirmed using CompuSyn software. Our observations showed that the combination indices of all the studied doses were <1, indicating a synergistic activity between the two agents. A lower combination index (denoting greater synergy) in both cell lines was observed for co-treatment with 0.5 IC50 celastrol and 0.5 IC50 5-FU (Table 2).

**Table 2:** Synergistic combination of celastrol with 5-fluorouracil (5-FU)

AGS			EPG85-257		
5-FU (μM)	Celastrol (μM)	CI	5-FU (μM)	Celastrol (μM)	CI
10	2	0.374	7	3.5	0.359
20	4	0.436	15	7	0.401
40	8	0.847	30	14	0.775

Note: The synergistic effect was evaluated using CompuSyn software. The anti-proliferative effect of 5-FU+celastrol at 0.5IC50, 1IC50, 2IC50 (10 + 2, 20 + 4 and 40 + 8 for AGS cells and 6 + 3.5, 12 + 7 and 30 + 15 μM for EPG85-257 cells) was measured using MTT assay. 5-FU: 5-Fluorouracil, CI: Combination Index.

### Effects of celastrol and 5-FU alone and their combination on apoptosis induction by flow cytometry

**Figure 2:**
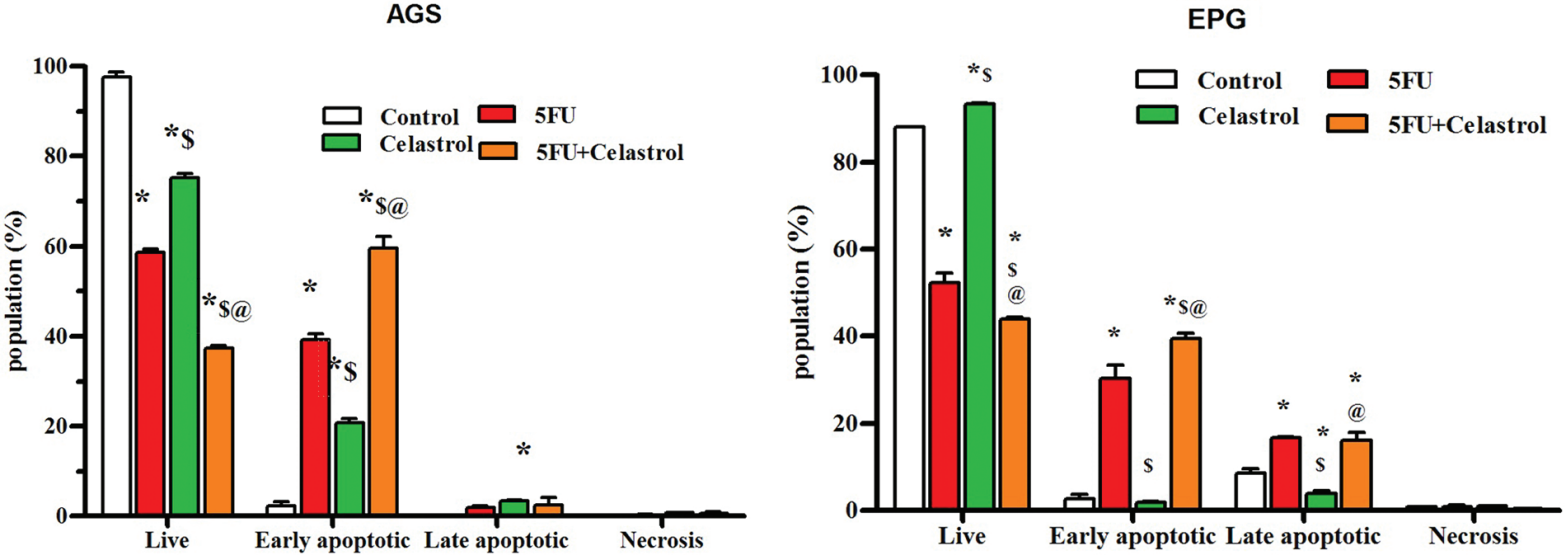
Cells were treated with the 0.5 IC50 of celastrol (2 μM for AGS cells and 3.5 μM for EPG cells), 5-FU (10 μM for AGS cells and 7 μM for EPG cells), and their combination for 48 h, and stained with propidium iodide (PI) and Annexin V-fluorescein isothiocyanate isomer (FITC), and then the rate of apoptosis was measured using flow cytometry; Data are presented as the mean ± SD of three independent experiments (*p < 0.05 *vs*. control group; ^$^*p* < 0.05 *vs*. 5-FU group; ^@^*p* < 0.05 *vs*. celastrol group).

The flow cytometry results showed that celastrol, 5-Fu, and their combination induced cell death due to apoptosis in the cell lines. The mean percentage of apoptosis for AGS cells treated with celastrol, 5-FU, and their combination was calculated at 23.9, 41.2, and 61.9, and for EPG85-257 cells at 5.65, 46.9, and 55.7, respectively. Apoptosis increased in co-treated cells with celastrol and 5-FU compared with the control (*p* < 0.05; Fig. 2).

### Effect of celastrol, 5-FU, and their combination on cell cycle inhibition

**Figure 3:**
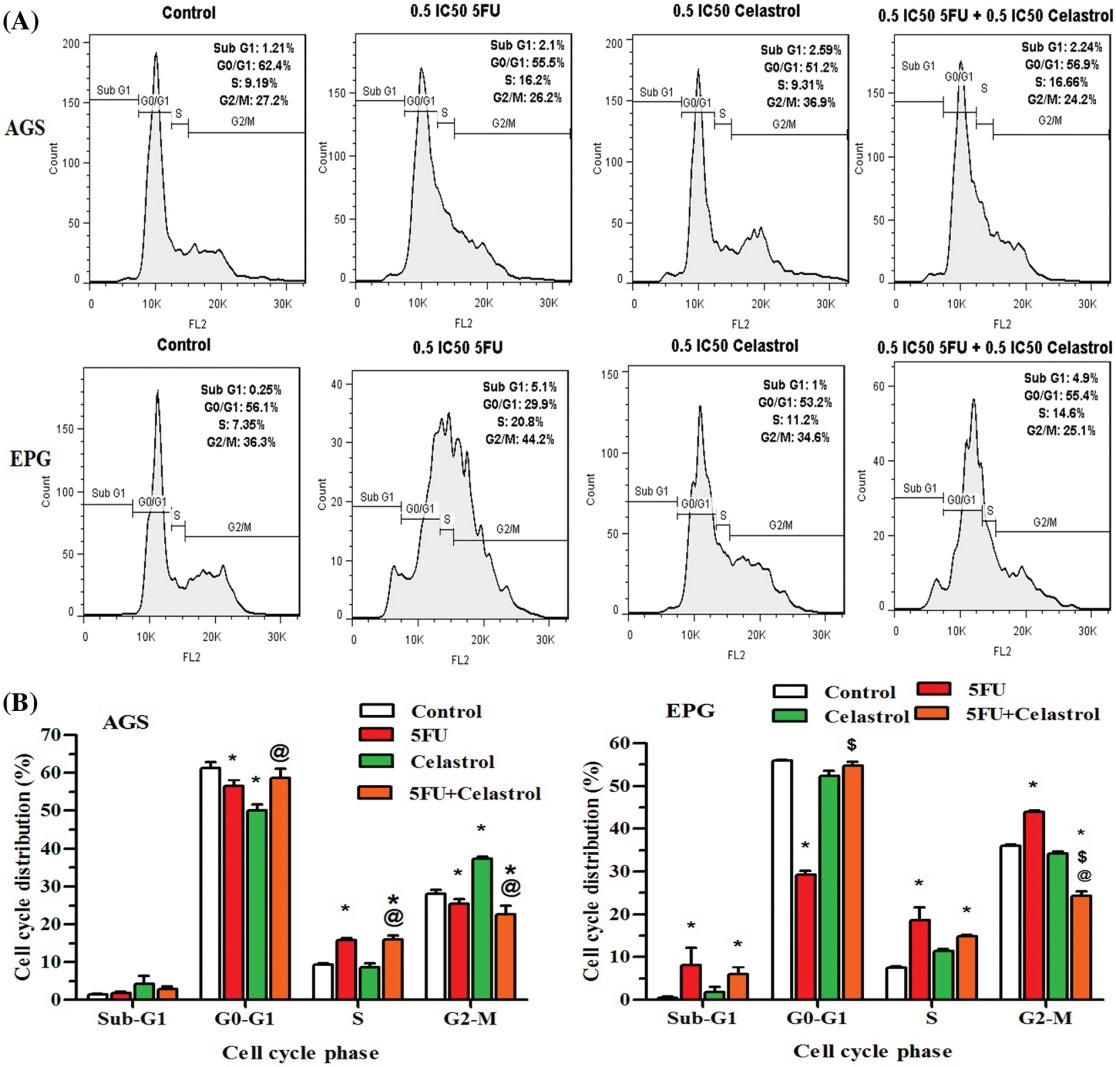
Cells were treated with 0.5IC 50 of celastrol (2 μM for AGS cells and 3.5 μM for EPG cells), 5-fluorouracil (10 μM for AGS cells and 7 μM for EPG cells), and their combination for 48 h and examined using BD Cycletest along with DNA reagent kit and flow cytometry; Data are presented as the mean ± SD of three independent experiments (*p < 0.05 *vs*. control group; ^$^*p* < 0.05 *vs*. 5-FU group; ^@^*p* < 0.05 *vs*. celastrol group).

The cells treated with celastrol, 5-FU, and their combination for 48 h showed a typical DNA pattern that represented sub-G1, G1, S, and G2/M phases of the cell cycle. The treated AGS cells with 10 μM (0.5 IC50) 5-FU increased the S population and decreased the G0/G1 and G2/M populations compared with the control group (*p* < 0.05). The AGS cells treated with 2 μM (0.5 IC50) celastrol increased the G2/M population and decreased the G0/G1 population compared with the control group (*p* < 0.05). Co-treatment of AGS cells with 10 μM 5-FU and 2 μM celastrol increased the S population compared with the control (*p* < 0.05) and celastrol (*p* < 0.05) groups. The EPG85-257 cells treated with 7 μM (0.5IC50) 5-FU increased the sub-G1, S, and G2/M population and decreased the G0/G1 population compared with the control group (*p* < 0.05). Co-treatment of EPG85-257 cells with 7 μM 5-FU and 3.5 μM celastrol increased the sub-G1 and S populations compared with the control group (*p* < 0.05) and increased the G0/G1 population and decreased the G2/M population compared with 5-FU (*p* < 0.05, Fig. 3).

## Discussion

The aim of this study was to investigate the effect of celastrol and 5-FU co-treatment on cell proliferation, cell cycle, and apoptosis induction in the AGS and EPG85-257 gastric cancer cell lines. The results of this study showed that co-treatment could increase the efficacy of the synthetic drug by inhibiting cell growth and inducing apoptosis along with reducing the dose of 5-FU.

In the present study, the anti-proliferative activity of celastrol on AGS and EPG85-257 cell lines was investigated. After co-treatment with celastrol and 5-FU, the cell viability significantly decreased in a dose-dependent manner. Some medicinal plants have been reported to produce anticancer effects by inducing programmed cell death [18]. The results of the present study showed that co-treatment of cells with 5-FU and celastrol could arrest the cell cycle at the synthesis phase and induce programmed cell death in early apoptosis. Celastrol is an immunomodulatory compound that produces anti-inflammatory effects by acting on the NF-κB and mitogen-activated protein kinase transcription factors [19]. The effects of celastrol have also been reported on tumor onset inhibition, tumor invasion, and metastasis in a wide range of tumor cells, as well as inhibitory impacts on cancer models *in vivo* [20]. In the study of Yang et al., celastrol isolated from Tripterygium inhibited the growth of breast cancer cells by inducing apoptosis [21]. Another study showed the efficacy of celastrol for inhibiting cell growth and proliferation and inducing cell death in the HL-60 cell line, prostate cancer, and glioma cells [22]. Ni et al. investigated the effect of celastrol on cell cycle arrest and apoptosis in human multiple myeloma and observed its impact on cell cycle arrest in myeloma cells [23]. Yoon et al. reported that celastrol caused the death of several cell lines of breast cancer and colon cancer by inducing apoptosis. In this study, they observed that celastrol increased mitochondrial calcium levels and dilated the endoplasmic reticulum by inhibiting proteasomes in the cell lines [24]. Celastrol can enhance the anticancer effect of gambogic acid by inhibiting NF-kB in squamous cell carcinoma cell lines [25]. As a potent proteasome inhibitor, celastrol has also been reported to induce apoptosis and cell cycle arrest in some cancer cells [26].

Raja et al. in a study on the anticancer activity of celastrol in combination with chemotherapy drugs for the treatment of human breast cancer, observed the efficacy of celastrol combined with chemotherapy drugs *in vitro* and *in vivo* [27]. These results are consistent with the results of the present study. In addition, it has been suggested that celastrol might be used as an effective anticancer agent to deal with drug resistance in lung cancer patients [28]. Celastrol has also been suggested as a promising new adjunctive therapy to treat hormone-resistant prostate cancer *in vitro* and *in vivo* [29]. Celastrol plays a role in the NF-κB pathway and produces an effect on this transcription factor through various mechanisms; the compound reduces the expression of IκB kinase and NF-κB, inhibits the NF-κB P60 subunit, and prevents its binding to DNA [23]. Also, it inhibits the enzyme by targeting cysteine 179 of the IKK kinase lope [30]. Therefore, it may decrease the expressions and concentrations of TNF-α and IL-6 in inflammatory diseases such as cancer [31,32].

Taken together, in the present study, celastrol could decrease the concentration of 5-FU required to suppress the AGS and EPG85-257 gastric cancer cell lines. As well, celastrol in combination with 5-FU induced cell cycle arrest and apoptosis. However, these observations deserve more and more research to contribute favorably to gastric cancer treatment.

## Data Availability

The data and materials used in the present study are available from the corresponding authors upon reasonable request.
